# Is Attention Based on Spatial Contextual Memory Preferentially Guided by Low Spatial Frequency Signals?

**DOI:** 10.1371/journal.pone.0065601

**Published:** 2013-06-11

**Authors:** Eva Zita Patai, Alice Buckley, Anna Christina Nobre

**Affiliations:** 1 Department of Experimental Psychology, University of Oxford, Oxford, United Kingdom; 2 Institute of Child Health, University College London, London, United Kingdom; 3 Oxford Centre for Human Brain Activity, University of Oxford, Oxford, United Kingdom; Cardiff University, United Kingdom

## Abstract

A popular model of visual perception states that coarse information (carried by low spatial frequencies) along the dorsal stream is rapidly transmitted to prefrontal and medial temporal areas, activating contextual information from memory, which can in turn constrain detailed input carried by high spatial frequencies arriving at a slower rate along the ventral visual stream, thus facilitating the processing of ambiguous visual stimuli. We were interested in testing whether this model contributes to memory-guided orienting of attention. In particular, we asked whether global, low-spatial frequency (LSF) inputs play a dominant role in triggering contextual memories in order to facilitate the processing of the upcoming target stimulus. We explored this question over four experiments. The first experiment replicated the LSF advantage reported in perceptual discrimination tasks by showing that participants were faster and more accurate at matching a low spatial frequency version of a scene, compared to a high spatial frequency version, to its original counterpart in a forced-choice task. The subsequent three experiments tested the relative contributions of low versus high spatial frequencies during memory-guided covert spatial attention orienting tasks. Replicating the effects of memory-guided attention, pre-exposure to scenes associated with specific spatial memories for target locations (memory cues) led to higher perceptual discrimination and faster response times to identify targets embedded in the scenes. However, either high or low spatial frequency cues were equally effective; LSF signals did not selectively or preferentially contribute to the memory-driven attention benefits to performance. Our results challenge a generalized model that LSFs activate contextual memories, which in turn bias attention and facilitate perception.

## Introduction

Memory is a fundamental mental faculty ever tuning our adaptation to the environment, and influencing perception and attentional processes directly [1]–[3]. Recently, we developed an experimental paradigm to investigate how long-term memory (LTM) can guide attention, and showed that the pre-exposure to a complex scene in which a target location had been learned modulates neural activity and facilitates behavioural responses to the subsequent appearance of the target at the remembered location [4]–[8]. Given this robust and well replicated memory-guided attention effect, we asked: what are the low-level visual mechanisms driving the memory signal?

The notion that fine perceptual discriminations are guided by feedback from high-order areas after an initial coarse (rapid and early) representation has been a prevalent notion in psychology [9]–[12]. Since Navon’s initial proposal of a global-to-local processing theory of vision [13], much of the research regarding visual processing has taken the approach that perhaps multiple streams of information run in parallel and influence one another, or alternatively are constructed in some hierarchical way in which different brain areas interact with different components of a visual image to construct a whole. A more recent model, the Reverse-Hierarchy-Theory [11] states that visual processing proceeds rapidly from the lower-level visual areas to higher-level prefrontal areas, and that feedback connections along this path are activated when more visual scrutiny is required. Specifically, the feed-forward process is automatic, and leads to a coarse, or global, representation of the visual input. As more detailed information is required, activation proceeds from prefrontal areas downward. This model can explain how identification of global properties is possible under sub-second exposures given the large receptive field properties in higher-order areas. One example would be the ability to discriminate the presence versus absence of an animal in a complex scene at very brief exposures ([14]. The model also proposes that re-activation of low-level areas can proceed in a serial fashion when required, as during effortful serial visual search [15]. This would indicate that vision at a glance is functionally equivalent to global precedence as proposed by Navon [13], and that this process is primarily the result of rapid feed-forward connections from early visual areas to higher-level prefrontal areas, which in turn trigger the ‘vision with scrutiny’ processes through feedback connections [11].

In light of these functional architectures, the general concept of coarse-to-fine processing has dominated the field of visual image processing [9], [10], [16], and has been extensively detailed and studied by Bar and colleagues using magnetic-resonance imaging and magnetoencephalography experiments [17]–[24]. The general idea is as follows: the visual system extracts both low and high spatial-frequency information from a visual input. These different sets of information are largely processed independently. The low spatial-frequency (LSF) information is primarily conveyed by magnocellular projections following the dorsal visual stream, and high spatial-frequency (HSF) information is mainly conveyed by parvocellular projections following the ventral visual stream. The rapid processing speed of the magnocellular pathway allows for information to reach higher-order areas such as the prefrontal cortex (PFC), which in turn bias the processing of HSF signals arriving along the slower parvocellular projections to the inferior-temporal cortex (IT) [16], [20], [21], [23]–[25]. Thus global, contextual information from re-entrant feedback connections can influence the slower feed-forward process of object identification. More recently, Bar has also proposed that the retrosplenial cortex (RSC) and parahippocampal cortex (PHC) are involved in the contextual guidance of scene processing ([20], [21], [23], [24]; but see [26] for opposing views). The advantage of such a system would be that predictive information from the environmental context could constrain the possible outcomes during the decoding of ambiguous input signals.

A few qualifications are worth mentioning before we accept the generalized model of coarse-to-fine visual-contextual processing. Though the distinction between a ventral and a dorsal stream of visual processing is useful for understanding the different aspects of visual perception and action [27], [28], it is not case that these two pathways are entirely segregated [29]. In addition, the general misconception that the dorsal pathway uses magnocellular projections and the ventral pathway is based on solely parvocellular input is much too simplified [10], [30], and LSFs and HSFs are not processed exclusively by magnocellular and parvocellular cells, respectively [16], [30]. Nevertheless, the model provides a simplified anatomically plausible and functionally well-documented framework for understanding how visual input is processed.

There have been studies that directly assess the contribution of the different visual pathways in the computation of natural image properties by using spatial filters, in order to separate the input into various visual processing channels. For example, it has been shown that LSF information is processed more rapidly and provides a ‘raw estimate’ for incoming HSF information, and that this effect is dependent on exposure times [31]. Specifically, at short exposures the LSF information was preferentially processed, whereas when longer processing time was available, HSF information was utilized [31]. Recently, using random-dot sterograms, it was shown that human pattern vision follows the coarse-to-fine order as well, indicating that this process starts from the basic visual input level, not just during scene-viewing [32]. In addition, it has recently been proposed that the neural signatures underlying global and local processing (which can be loosely equated with low and high spatial-frequency processing) can be separated: low frequency oscillation in the theta band corresponds to global information processing, while higher frequency beta band activity underlies local processing [33], [34]. All these findings converge to support the coarse-to-fine hypothesis, namely that activity from higher-order areas may precede and enhance neural activity in early visual cortices; it is on this premise that we conducted a set of experiments to test whether LTM benefits to perception depending on contextual signals is carried by a coarse-to-fine mechanism.

The different proposals of how such a mechanism may work are indicative that the brain is no longer seen as a passive computing device, but is instead actively involved in selecting and modulating incoming information. In this case, an internal signal, such as memory, could plausibly interact with incoming information directly. Memory guided attention is not only a robust effect in magnitude, but also very rapid, being firmly established by 100 ms lead time [5]. Thus, we were interested in whether the mechanisms of rapid scene perception are invoked during memory-guided attentional orienting, specifically whether memory-guided biases are selectively driven by coarse visual representations.

The basis for our experiments was the coarse-to-fine model of scene recognition, and the assumption that contextual information coming from MTL areas should further boost the LSF effect, especially if the contextual memories are highly relevant for a difficult discrimination task. Given that the contextual-guidance model proposed by Bar involves medial-temporal areas (MTL) [20], [21], [23], [24], which are typically associated with spatial navigation and/or memory processes [35]–[39], we manipulated directly the low and high spatial-frequency information that activates the contextual memories in order to test for the anticipated LSF advantage in behaviour relating to scene processing. We hypothesized that if LSFs are faster at guiding scene recognition, they should also be quicker at activating relevant contextual memories, thus facilitating target selection in a previously memorized location.

Experiment 1 was a control scene-perception task designed to ensure that the stimulus and task parameters were appropriate for replicating the well documented LSF advantage (for early example see [31]). Filtered scenes containing only low or high spatial-frequency information were presented very briefly (two refresh rates on a 60 Hz screen) followed by a choice of two scenes. Participants made a forced-choice discrimination. Once the LSF advantage was clearly replicated, it was possible to use the filtered images as memory cues in the memory-guided attention task we have developed [5].

In all the following experiments (Exp. 2–4), there was a learning phase, during which participants learned specific context-target associations, followed by a perceptual discrimination task, in which the cue scene preceded the presentation of the target to be identified. The cues were filtered in order to provide only low or high spatial-frequency information. The main experimental question of interest was whether the top-down memory signal biasing perception during memory-guided orienting is comprised primarily of LSF signals, acting in a way that is analogous to the top-down feedback signals during natural scene perception. If so, activation of memory cues using LSF stimulation should trigger memory biases that can be established more quickly and which can act more effectively than HSF stimulation.

## Materials and Methods

### Stimuli

For the following experiments, the stimuli used were digital photographs of scenes filtered to contain either low (LSF) or high spatial-frequency (HSF) information only. All scene stimuli were created from photographs obtained collectively by the lab. Images contained indoor environments, cityscapes, or landscapes, without any conspicuous human characters or animals. All images were converted to greyscale, and resized to 1000×750 pixel images using Matlab (Mathworks, Natick, MA). They were filtered using a Gaussian filter, with a cut-off frequency of 2 cpd (cycles per degree) for low spatial-frequency images (keeping all frequencies below this value), and 6 cpd for high spatial-frequency images (keeping all frequencies above this value). These cut-off values are typical for filtering images [31], [40], and provide a distance and image-size independent measure of spatial frequency.

Luminance values were tested with a customized Matlab protocol, which used saturation values in the red, green and blue channels to estimate luminance. This step was implemented to ensure that the behavioural effects relating to the spatial frequency filtering were not overshadowed by other low-level differences of perceptual saliency in the images, which result from the filtering process itself. Luminance values of the filtered scenes were extracted and tested for differences with two-sample independent t-tests, which were found to be non-significant (comparing non-filtered to LSF, t(286) = –.52; p = .61; non-filtered to HSF, t(286) = –.06; p = .95; HSF to LSF, t(286) = .33, p = .74).

### Ethics Statement

All participants were volunteers recruited from a subject pool at the University of Oxford, and gave written consent to participate in this study for monetary compensation. The studies were approved by the University of Oxford Central University Research Ethics Committee (CUREC).

## Experiment 1

In order to confirm that the stimuli were suitable for the subsequent experiments, a short scene discrimination task was used. Participants viewed a filtered image presented for two refresh rates (33 ms for the 60 Hz monitor used), which, after a short inter-stimulus interval (33 ms), was followed by a display of two images, one matching the probe stimulus, the other a foil. The task was to indicate which of the two images matched the filtered sample scene. We expected an advantage for low spatial-frequency sample scenes, borne out by faster reaction times and higher identification accuracy. The reasoning behind using very short exposure durations was to maximize the advantage of fast processing speeds usually observed for LSF stimuli [31].

### Methods

#### Participants

Twelve volunteers (11 females, mean age: 19 years, 1 left-handed) participated in this study.

#### Scene stimuli

Ninety six greyscale scenes were used in the experiment.

#### Procedure

**Figure 1:**
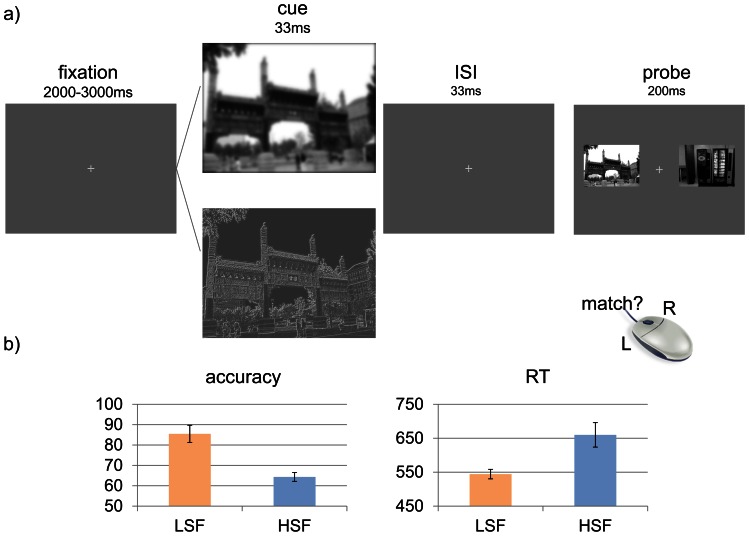
Experiment 1 task design and results. a) Trial sequence in the perceptual choice task. A jittered pre-trial fixation was followed by one of two types of image: low or high spatial- frequency filtered sample scene. This was followed by an ISI of 2 refresh rates, and finally the probe images, which were never filtered. Participants had to indicate with a mouse press which of the two images matched the preceding filtered sample (left mouse button for left-sided match, right mouse button for right-sided match). b) Results showed RT and accuracy benefits for probes preceded by LSF filtered sample scenes (error bars represent standard errors).

Participants performed 96 trials in which a HSF or LSF sample scene appeared briefly (33 ms, subtending a visual angle of 19.9°×14.9°) and was followed shortly afterward (inter-stimulus interval – ISI - of 33 ms) by a probe array containing two full-greyscale scenes (200 ms, subtending a visual angle of 8.3°×6.5°, on either side of fixation). One of the scenes in the probe array matched the filtered sample scene and one was a novel scene. Participants made a speeded forced-choice response indicating which of the two probe scenes matched the previous filtered sample scene (Figure 1a) using a mouse-click (left mouse button if scene on left matched the previously presented filtered scene, right mouse for right-sided match). They were instructed to respond as quickly and as accurately as possible (Figure 1a). Trials containing HSF and LSF scenes appeared in a random order, and assignment of each scene to the HSF or LSF condition in the sample and to side of presentation in the probe array was counterbalanced across subjects. No feedback was given, and participants had 1000 ms to respond. The inter-trial-interval was jittered between 2 and 3 seconds.

### Results and Discussion

Reaction times to identify the sample scene were recorded, and accuracy scores calculated (percent correct). A paired-samples t-test was used to assess the differences between the mean RTs and accuracy scores of the different spatial frequencies. Low spatial-frequency images resulted in significantly more accurate, t(11) = 5.34, p<.001 (two-tailed), and faster, t(11) = 3.49, p = .005 (two-tailed), responses. Figure 1b shows the mean performance on the choice task.

In this experiment, the typical finding of a LSF advantage during the rapid perceptual categorization of natural scenes (for example: [31], [41]) was replicated, thus confirming that the stimuli are appropriate for use in subsequent experiments.

## Experiment 2

The main purpose of the subsequent experiments was to test whether long-term memory (LTM) biases on perception are primarily or selectively activated by rapid, LSF information. In Experiment 2, we tested whether the spatial-frequency memory-cues would modulate subsequent target processing differently from memory-cues containing the full image information (no filter). Participants performed a memory-guided perceptual discrimination task, which consisted of a learning phase and a memory-guided attention phase. Participants performed these two experimental phases over three days. Over the first two days, they completed a Learning Task, in which they explored visual scenes to learn the location of a target (a small gold key) in each scene (50% of scenes contained a key). By the end of the learning task, participants had formed strong spatial contextual memories of the target location for scenes containing a target, but they had no specific target-context associations for those scenes that did not contain a target (all scenes however, were familiar).

On the third day, they completed a memory-guided attention Orienting Task in which they discriminated the presence or absence of a target (also a small gold key) embedded within a full greyscale scene. Pre-exposure to a filtered version of the scene (without any target) provided memory-based cues to orient contextual spatial attention to the location of the remembered target.

If the contextual memories formed during learning are activated more quickly and/or more strongly when they are driven by only LSF information, then we would expect to find a greater behavioural benefit in reaction times and accuracy after pre-exposure to filtered cues containing LSF compared to HSF information. This would be borne out by an interaction between the effects of spatial memory carried by the cue and the spatial-frequency of the cues (i.e. valid LSF memory cues should facilitate attentional processes and lead to better behavioural performance than HSF memory cues, or neutral cues with no specific memory associations for the target location).

### Methods

#### Participants

Power calculations based on Experiment 1 and on our previous memory-guided orienting study using a similar paradigm [8] showed that a minimum of 6–10 participants was required to reveal significant orienting effects. The number of participants in this and subsequent experiments was determined by the number of participants required to counterbalance all relevant experimental factors. Twenty-four volunteers (5 male, 19 female, mean age = 24 yrs) participated in this study.

#### Scene stimuli

One hundred and forty four greyscale scenes were used in the experiment. For the learning task, a small key (size: 0.5 cm×1 cm, subtending a visual angle of 0.25°×0.50°) was placed in one of the four quadrants of the scene, preferably in a hidden location (the key looked like a typical door lock key, oriented vertically upwards). Five versions of each learning-task display were generated for each scene, with the key placed in one of each of the four quadrants or with the key absent – this was done for counterbalancing purposes. For the orienting task, the scenes with keys were re-made to include a larger and brighter key (size: 1 cm×1.8 cm, subtending a visual angle of 0.5°×0.9°) in the location of the original key target. Two additional types of scenes, with a filter, were prepared for the orienting task.

#### Learning task

Participants viewed each of the 144 scenes in a random order, repeated over six blocks (the learning task was broken down into three blocks each, over two consecutive days). Half of the scenes contained a small key target in one of the four quadrants. The remaining 72 scenes did not contain a target. Participants viewed the scenes and searched for the target overtly. Once located, participants clicked once with the mouse to activate a cursor, after which they clicked on the location of the key with the mouse. After a response, or after available search time expired, the next scene was presented. The available search time decreased as the blocks progressed, with the maximum duration of each scene randomized within a range (16–24 s in block 1, 12–20 s in blocks 2 and 3, 10–18 s in blocks 4 and 5, 8–16 s in block 6). Exposure times for key-absent scenes were yoked to the exposure of key-present scenes. Participants had to find as many keys as possible and memorize their locations. Eye movements were recorded using an infrared eye-tracking system (ISCAN, Woburn, MA). Only participants that located more than 80% of keys in target-present scenes progressed to the next phase of the experiment.

#### Orienting task

**Figure 2:**
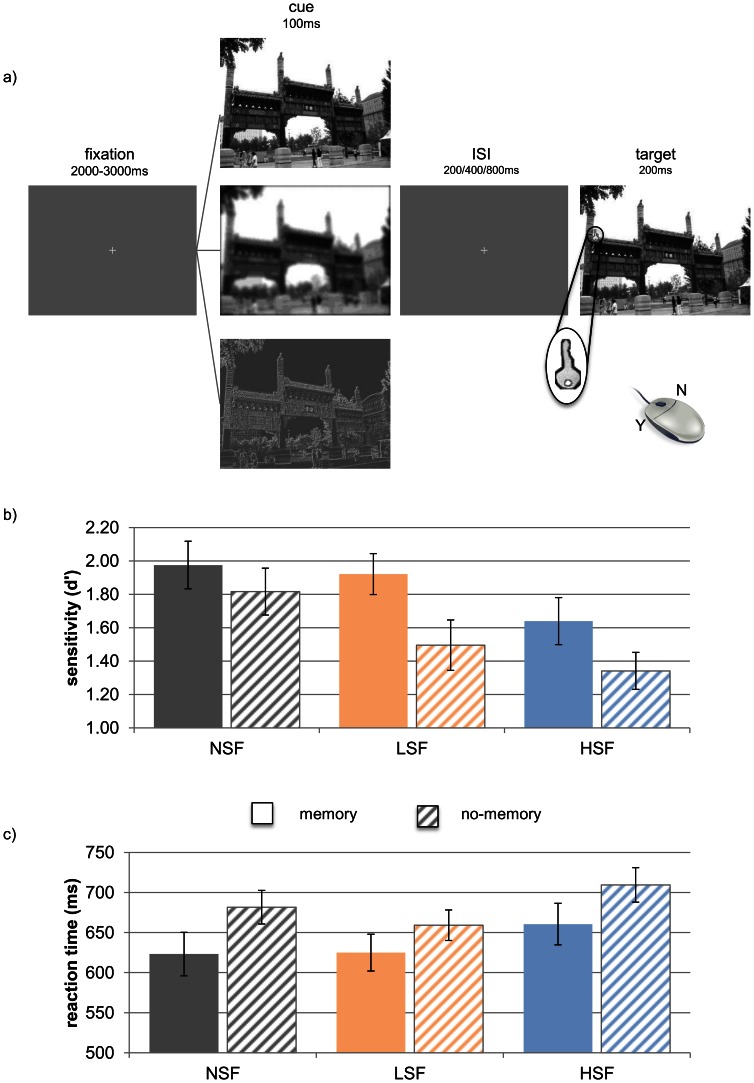
Paradigm and Results of Experiment 2. a) Trial sequence in the orienting task. A jittered pre-trial fixation was followed by one of three types of cue: non-filtered, low- or high-spatial-frequency filtered image. This was followed by a variable inter-stimulus interval, and finally the target image, which was never filtered. Participants had to indicate with a mouse press whether or not there was a target currently present in the target image. b) Sensitivity scores and c) reaction times (for target present trials only) for each cue type (NSF, LSF, HSF) by memory condition (memory, no-memory). Error bars represent standard errors.

Participants performed 144 trials. The task was to detect, using covert attention, the presence or absence of a bright key within the familiar scenes that had previously been studied. Each trial began with the presentation of a familiar scene (100 ms), which was used to cue the participant’s attention to a particular location within the scene. This cue contained no embedded key target, and could be presented in one of three conditions: normal (NSF, unfiltered), low spatial-frequency (LSF), or high spatial-frequency (HSF) (Figure 2a). After a variable ISI (200, 400 or 800 ms), the probe scene appeared (200 ms), with or without a target embedded. The probe scene was never filtered. Participants indicated with a mouse button press whether a target was present in the probe scene (left button: target present; right button: target absent). They were instructed to respond as quickly as possible but to avoid making mistakes.

The design crossed the factors of spatial-frequency of cue (NSF, LSF, HSF), spatial memory (memory, no-memory), and target presence (present, absent). There were twelve trials in each cell.

### Results and Discussion

The total number of subjects included in the analysis was twenty two (two subjects were excluded: one participant failed to locate at least 80% of targets in the learning task, and one participant was excluded for having a d’ score that was more than 2.5 standard deviations below the mean.

#### Learning task

Search times were calculated as time from scene onset to the time that the subject made their first mouse click (to activate the cursor). As the learning blocks progressed, reaction times decreased and more targets were located (block 1 mean accuracy = 65%±3.7 SEM, mean search times = 6 s±0.4; block 6 mean accuracy = 83%±4.2; mean search times: 1.5 s±0.2) Repeated-measures ANOVAs testing for linear decreases in reaction time and linear increases in accuracy over the learning blocks revealed significant linear contrasts for both measures (reaction time: F(1,21) = 118.17, p<.001; accuracy: F(1,21) = 369.59, p<.001).

#### Orienting task

Support for the hypothesis that magnocellular signals guide contextual cueing by LTM would be borne out by an interaction between memory and spatial-frequency. A repeated-measures ANOVA of d’ revealed significant main effects of spatial-frequency (F(1,21) = 5.39, p = .008) and memory (F(1,21) = 12.89, p = .002). Perceptual discrimination scores were higher for memory trials; and performance in normal (NSF) and low spatial-frequency conditions (LSF) was better than in the high-spatial-frequency (HSF) condition (Figure 2b). However, critically, no interaction was observed between these two factors (F(1,42) = .614,p = 0.55).

A repeated-measures ANOVA on reaction times (Figure 2c) revealed significant effects of SF (F(1,42) = 16.85, p<.001), with LSF cues resulting in faster RTs. In addition, there was a significant effect of,target presence (F(1,21) = 28.75, p<.001), and an interaction between memory and target presence (F(1,21) = 42.1, p<.001). Participants were faster to respond in target-present trials, especially when they had a memory for the target location. Again, there was no interaction of spatial-frequency and memory (F(1,42) = 2.51, p = .78). No other main effects or interactions were significant (all p>.1). When looking at reaction times relating to target-present trials only, there was a significant effect of SF (F(1,21) = 7.492, p = .002), and memory (F(1,21) = 14.357, p = .001), but no interaction (F(1,42) = .431, p = .65). Detailed p-values for each condition and interaction, along with effect sizes are available in Table S1.

Inverse efficiency scores (RT/accuracy) showed a significant effect of SF (F(2,42) = 8.895, p = .001), memory (F(1,21) = 15.907, p = .001), a trend for target presence (F(1,21) = 3.879, p = .062), and an interaction between memory and target presence (F(1,21) = 41.774, p<.001). But again no interaction occurred involving memory and spatial frequency (F(1,21) = .550, p = .581).

The findings of this experiment show that the memory-based attentional guidance observed in previous reports (for example see: [5]) can be replicated even when using cues with limited spatial-frequency information However, the lack of interaction between the spatial-frequency and memory factors suggests that either these two mechanisms operate independently, or our experiment was not sensitive to this interaction.

## Experiment 3

In this experiment, the aim was to probe further for the potential interaction, by reducing the number of conditions.

### Methods

Sixteen healthy students (10 male, mean age = 22yrs, 3 left-handed) participated. The stimuli and training procedures were the same as in Experiment 2, except 160 scenes were used. The orienting task was the same as in Experiment 2, except for two differences: (1) the unfiltered cue scenes were removed, leaving only two spatial-frequency cue conditions: LSF and HSF, (2) only two ISIs were used, one short (100 ms) and one long (700 ms). The design crossed the factors of spatial-frequency of cue (LSF, HSF), spatial memory (memory, no-memory), target presence (present, absent), and ISIs (100, 700), resulting in ten trials in each cell.

#### Spatial memory recall task

In order to get an approximate measure of the state of recollective memory in the session, following the orienting task, participants performed a recall task that measured explicit memory for target locations. Participants viewed all 160 scenes (greyscale, but unfiltered, as in learning task), without any targets. For scenes in which they had a memory for the target location, they used the mouse to click on the remembered target location from the learning phase. If they had no memory, they were instructed to click the centre of the screen. Participants were also instructed to rate their confidence in their responses after each scene by clicking one of the three mouse buttons to indicate strength of confidence (range: not at all confident, fairly confident, and very confident).

### Results and Discussion

#### Learning task

As the learning blocks progressed, reaction times decreased and more targets were located (block 1 mean accuracy = 63%±4.7SEM, mean search times = 6.6 s±0.29; block 6 mean accuracy = 87.8%±5.7; mean search times: 1.8 s±0.13). Repeated-measures ANOVAs testing for linear decreases in reaction time and linear increases in accuracy over the learning blocks revealed significant linear contrasts for both measures (reaction time: F(1,15) = 187.35, p<.001; accuracy: F(1,15) = 103.71, p<.001).

#### Orienting task

**Figure 3:**
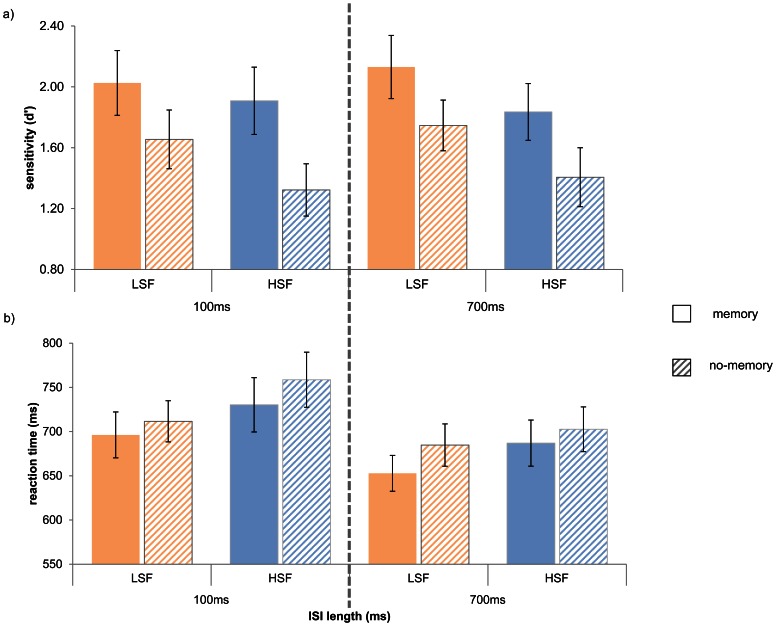
Results of Experiment 3. a) Sensitivity scores and b) reaction times (for target present trials only) for each cue type (LSF, HSF) by memory condition (memory, no-memory), grouped by ISI (100 ms, 700 ms). Error bars represent standard errors.

A repeated-measures ANOVA of d’ (Figure 3a) revealed a significant main effect of memory (F(1,15) = 20.38, p<.001). Perceptual discriminations were higher when cues carried memory for the target location. No other significant main effects (spatial-frequency (F(1,15) = 2.78, p = .12), ISI (F(1,15) = .149,p = 0.71)) or interactions (all p>.1) were observed. The interaction of interest, between memory and spatial frequency was far from significant (F(1,15) = .284, p = 0.6), as was the three-way interaction of memory, spatial frequency and ISI (F(1,15) = .182, p = 0.7).

A repeated-measures ANOVA on reaction times (Figure 3b) revealed participants were faster at responding to LSF cues (F(1,15) = 19.79, p<.001), target present trials (F(1,15) = 35.26, p<.001), and trials where the ISI was shorter (F(1,15) = 16.13, p = .001). There was an interaction between memory and target presence (F(1,15) = 14.92, p = .002), but no main effect of memory (F(1,15) = .73, p = .41), and no interaction between spatial-frequency and memory (F(1,14) = .01, p = .93). No other interactions were significant (all p>.1). When looking at reaction times relating to target-present trials only, there was a significant effect of SF (F(1,15) = 6.15, p = .026) and ISI (F(1,15) = 9.28, p = .008 ), a trend towards an effect of memory (F(1,15) = 4.263, p = 0.057), but no interaction between memory and spatial frequency (F(1,15) = .007, p = .933), and no three-way interaction (F(1,15) = 0.63, p = 0.44), and no other significant interactions (p>.1). Detailed p-values for each condition and interaction, along with effect sizes are available in Table S1.

In addition, inverse efficiency scores (RT/accuracy) were used to analyse results independent of any possible speed-accuracy trade-offs. Analysis of inverse efficiency yielded significant effects of SF (F(1,14) = 5.73, p = .031), memory (F(1,14) = 6.31, p = .025), and a trend towards target presence (F (1,14) = 5.327, p = .081); as well as interactions between memory and target presence (F(1,14) = 11.105, p = .005); among spatial-frequency, target presence, and ISI (F(1,14) = 4.823, p = .045); and a trend for memory, target presence and ISI (F(1,14) = 4. 286, p = .057). Again, no interactions involving memory and SF approached significance (SF×memory (F(1,15) = 2.0, p = .18), SF×memory×ISI (F(1,15) = 1.4, p = .25), SF×memory×target presence (F(1,15) = .41, p = .53)).

#### Spatial memory recall task

In order to obtain a rough estimate of participants’ explicit memory for the target location, the number of scenes was calculated in which participants placed the key within a 150-pixel diameter circle around the original target location (approximately 3.4°visual angle/15% of screen). This calculation was performed only for trials that were target-absent in the orienting task, in order to avoid any contamination effects from being re-exposed to the target locations. The majority of subjects correctly identified the locations of the learned targets (group mean correct = 67±17% significantly different from chance (t = 3.8, p = 0.002)). In addition, participants’ confidence increased proportionally with their accuracy, which was measured by the distance between the remembered location and actual location of the key (mean distance from original target location of confidence rating 1– not at all confident = 68 pixels±4 SEM, rating 2– fairly confident = 66 pixels±6 SEM, rating 3– very confident = 40 pixels±2.5 SEM). A repeated-measures ANOVA testing for linear decreases in pixel distance from original key location over confidence ratings revealed significant a linear contrast (F(1,7) = 9.37, p = .018).

The results of this experiment show that, even when correcting for possible trade-offs in speed and accuracy, and separating short versus long ISIs, the effects of spatial-frequency and of memory are not accompanied by an interaction between these factors. LSF cues and memory cues each independently result in faster reaction times, but when combined do not offer an added benefit, as indexed by the lack of interaction. Memory cues also lead to higher perceptual discrimination, but again independently of any interaction with spatial frequency. One remaining important possibility to test was whether interaction between memory and spatial frequency would only be unveiled under even shorter duration exposures for the filtered cue scenes, and cue-target stimulus-onset asynchronies. Perhaps a selective LSF-driven memory effect only occurs before there has been time to invoke analysis of fine details in the slower HSF pathway. To test for this, in the last experiment of the series, the durations of cue and SOA were reduced to the values used in Experiment 1.

## Experiment 4

Experiment 4 used a very short cue duration and cue-target interval in order to test whether LSF signals play a prevalent role in memory-guided contextual cueing early on. The design was identical to that of Experiment 3, except for the stimulus timings: the cue and ISI were both at two refresh rates (33 ms). These values were chosen based on Experiment 1, which demonstrated a behavioural advantage for perceptually driven contextual priming at these intervals.

### Methods

Twenty-one students (6 male, mean age = 22 yrs, 2 left-handed) participated. The stimuli and training procedures were the same as in Experiments 2 and 3, except that 96 scenes were used. As a result, the six blocks were considerably shorter, and therefore training was conducted in a single two hour session.

#### Orienting task

The orienting task was the same as in Experiment 3, except that the exposure time of the cue and ISI were changed to be 2 refresh rates each (33 ms). The full factorial design included the factors of spatial-frequency of cue (LSF, HSF), spatial memory (memory, no-memory), and target presence (present, absent).

#### Spatial memory recall task

The subsequent test for recall of the spatial position targets within scenes used the identical procedure as Experiment 3.

### Results and Discussion

#### Learning task

As the learning blocks progressed, reaction times decreased and more targets were located (block 1 mean accuracy = 70%±2.7SEM, mean search times = 6.7±0.2 SEM; block 6 mean accuracy = 89%±1.5; mean search times: 1.3 s±0.1). Repeated-measures ANOVAs testing for linear decreases in reaction time and linear increases in accuracy over the learning blocks revealed significant linear contrasts for both measures (reaction time: F(1,20) = 468.32, p<.001; accuracy: F(1,20) = 225.06, p<.001).

#### Orienting task

This final experiment reduced exposure times in an attempt to isolate the early effects of LSF processing, and its potential contribution to relaying top-down memory-related signals to facilitate perception. A repeated-measures ANOVA of d’ (Table 1) revealed a trend for spatial-frequency (F(1,20) = 3.028, p = 0.097), but no effect of memory (F(1,20) = .373, p = .548). The interaction of interest between these two factors was also far from significant (F(1,20) = .024, p = 0.879).

**Table 1:** Results from Experiment 4.

Condition	Reaction Time (ms)	Sensitivity (d’)
LSF memory	680 (22)/855 (24)	1.85 (0.16)
LSF no-memory	687 (19)/806 (28)	1.90 (0.13)
HSF memory	696 (22)/887 (26)	1.63 (0.12)
HSF no-memory	723 (19)/821 (23)	1.72 (0.12)

For reaction time, data is shown for target present and absent trials separately, with target present values presented on the left, target absent on the right. Values in parentheses denote standard error.

A repeated-measures ANOVA on reaction times (Table 1) revealed significant main effects of SF (F(1,20) = 9.04, p = 0.007), memory (F(1,20) = 5.508, p = 0.029), and target presence (F(1,20) = 48.7, p<0.001). Responses were faster in trials with LSF cues, in trials with valid memory cues, and in target-present trials. There was no significant interaction between spatial frequency and memory (F(1,20) = .007, p = .932), or for the three-way interaction of memory (F(1,15) = .029, p = 0.87), or spatial frequency and target presence (F(1,15) = 2.08, p = 0.17). Only the interaction between memory and target presence was significant (F(1,20) = 14.29, p = 0.001). Detailed p-values for each condition and interaction, along with effect sizes are available in Table S1.

A repeated-measures ANOVA on inverse-efficiency scores revealed a significant effect of spatial-frequency (F(1,20) = 5.312, p = .032) and no other main effects (all p>.1). An interaction between memory and target presence (F(1,20) = 16.423, p<.001) also occurred. Post-hoc analysis showed that this interaction was driven by the fact that memory facilitated identification of key presence but tended to interfere with correct rejection of key absence (Table 1).

Again, there was no interaction of spatial-frequency and memory (F(1,20) = .223, p = .642), however a trend towards a three-way interaction of spatial-frequency, memory, and target presence was observed (F(1,20) = 3.195, p = 0.089). Given the potential relevance of this effect to the experimental hypotheses, subsidiary ANOVAs were used to characterise the nature of this trend. A 2×2 ANOVA on spatial frequency and memory focusing on target-present trials revealed a trend towards spatial frequency (F(1,20) = 3.8, p = 0.06), a significant effect of memory (F(1,20) = 6.65, p = 0.018), but no interaction (F(1,20) = 1.48, p = 0.24). In target-absent trials, the effect of spatial frequency was no longer significant (F(1,20) = 1.64, p = 0.22), however there was a significant effect of memory (F(1,20) = 14.9, p = 0.001), but again not interaction (F(1,20) = 0.73, p = 0.41). We can conclude from this analysis that in the three-way interaction the spatial frequency effect observed was driven by the presence of the target, while memory effects were consistent. These results further corroborate previous evidence that spatial frequency and memory do not interact in this task.

#### Spatial memory recall task

Performance in spatial memory recall task was calculated as described in Experiment 3. The majority of subjects correctly identified the locations of the learned targets (group mean correct = 78%), and confidence increased proportionally with the distance between the remembered location and actual location of the key (mean distance from original target location of confidence rating 1– not at all confident = 60 pixels±8.1 SE, rating 2– fairly confident = 51 pixels±4.4 SE, rating 3– very confident = 37 pixels±1.8 SE). A repeated-measures ANOVA testing for linear decreases in pixel distance from original key location over confidence ratings revealed a significant linear contrast (F(1,19) = 9.15, p = .007).

The results of this experiment do not provide any evidence for prevalent effect of LSF in carrying memory signals. The trend towards a three-way interaction of spatial-frequency, memory and target presence is a potential indication that a simpler task, such as a detection task may be more appropriate for probing the spatial-frequency and memory interaction at such short exposure durations.

## Bayesian Null-Hypothesis Testing

Bayesian null-hypothesis testing is an alternative to traditional null-hypothesis significance testing, allowing for a way of generating a graded level of evidence regarding which model (null or alternative hypothesis) is more strongly supported by the data [42], [43]. We used a simple formula available from Masson [42], which is calculated from the user input of: number of independent observations, degrees of freedom error, sum of squares effect and sum of squares error. This formula is based on the Bayesian probability theory, which takes into account the a priori probability of the hypothesis being true and the probability of obtaining the observed data independent of any hypothesis, resulting in posterior probabilities of both the null (H_0_) and alternative (H_1)_ hypothesis (as opposed to NHST where a binary decision is made whether to favour the H_0_ or H_1_ based on a cut-off value of p = 0.05). We used this formula to test the absence of the interaction effect between spatial frequency and memory observed in the data, over the three experiments (Experiment 2,3 and 4), in order to determine whether the lack of effect can be explained by support for the null hypothesis. The data presented in the Table 2 show the values in support of the null and alternative hypotheses, where the closer a number is to one, the more the associated hypothesis is supported by the data, with any number over 0.75 being positive evidence for the given hypothesis [44]. The data clearly show that using this method, we are able to provide secondary, numerical support favouring the null hypothesis, i.e. no interaction between spatial-frequency and memory signals.

**Table 2:** Results from the Bayesian null-hypothesis testing.

	Reaction Times		d’ (Sensitivity)	
	H_0_	H_1_	H_0_	H_1_
Experiment 2	**0.99**	1.44E-06	**0.96**	0.04
Experiment 3	**0.87**	0.13	**0.82**	0.18
Experiment 4	**0.89**	0.11	**0.88**	0.12

Evidence for both the null and alternative hypothesis for the spatial-frequency by memory interaction are presented for all three experiments.

### General Discussion

The goal of this set of experiments was to explore whether manipulating the spatial-frequency information available during the cueing period could modulate memory-guided attention. Given the coarse-to-fine hypothesis of visual processing, and the model of contextual facilitation in object perception [21], [24], we expected to find a greater benefit for memories that were cued by LSF information, as opposed to HSF. Behaviourally this would be borne out by an interaction between spatial-frequency and memory. However, neither sensitivity scores nor reaction times provided any evidence for a privileged or dominant role of LSF in carrying memory-based contextual cueing effects. There are many possible reasons for this.

Firstly, it is possible that there was something amiss with the stimuli used. This is unlikely. Experiment 1 confirmed the expected LSF advantage when participants had to match a probe to one of two target scenes, a task which is commonly used in the literature. Additionally, basic properties of the filtered images themselves may contain information that is different, leading to a benefit of one stimulus type over another [45]. In a series of experiments, Rotshtein and colleagues found conflicting evidence of spatial-frequency usage, so they carried out an analysis of low-level stimulus properties and found that the main diagnostic element was orientation information. Moreover, this information could explain why certain stimuli were preferred in one spatial-frequency in one task, but not in another [45]. In their task, stimuli within categories (house or flower) usually had similar orientation information, which could be diagnostic for task performance. In contrast, in the current tasks the stimuli were pictures of indoor and outdoor scenes, and the general make-up of diagnostic information was similar. In addition, assignment of stimuli across the experimental conditions of interest was counterbalanced across participants. It is therefore safe to conclude that the differential role of LSF and HSF information in the stimuli did not cause the lack of effects. Additionally, we replicated the LSF benefits previously shown in the literature; however, these just did not interact with our memory manipulation.

The second possible explanation relates to the task design. Over the course of the three memory-guided experiments, various effects of spatial-frequency and memory, as well as other factors, were observed. It is possible that the interaction of spatial-frequency and memory was overshadowed by the difficulty of the task or the low number of trials in the conditions. The former is most probably not the case, as the inverse efficiency scores show the same pattern of data, indicating that the interaction could not have been masked by poor or biased performance. Problems of statistical power were addressed by condensing the design in Experiment 3 and Experiment 4, to include a greater number of trials per condition. The consistency of the pattern of results across the multiple experiments, using both traditional and Bayesian null-hypothesis significance testing, also speaks for the reliability of the data.

Additionally, it is possible that participants were not using the cues enough to trigger a spatial-frequency by memory interaction. Since the targets were always embedded in the given context (which contained the relevant memory-related information), the cue may not have been necessary to perform the task. This criticism can be dismissed, because there were reliable effects of memory, as well of spatial-frequency, which would indicate that the information in the cues did influence the processing of the upcoming target stimulus.

It may be informative to run an experiment where only the cue contains spatial-frequency/memory-related information. Perhaps given that fine discriminations need to be made in order to separate the target form the background, HSF signals are just as important, and therefore the interaction of spatial frequency and memory is masked by the nature of the task. An alternative would be to present the target on a blank background, after a filtered cue scene, which could provide a memory-based spatial cue (context) as well as an opportunity to observe the effects of the different frequencies present in the cue. The problem with this alternative approach is that it does not rule out the confound that the target selection itself may operate independently (on HSF signals) from attentional guidance, which may or may not be selectively facilitated by LSF cues. Given the subsidiary analyses performed on the results of this task, it seems that the spatial frequency signals and the memory-driven attention effect are largely independent. Further studies are needed to separate the cueing effects from target-in-context effects.

Accepting the pattern of results across our experiments as representative, it is worth re-evaluating the hypothesis and models upon which the experiments were based. The coarse-to-fine model states that LSF information is processed more quickly, mainly due to it being carried by magnocellular pathways, and it thus provides a coarse representation of the visual input sufficient for processing its general attributes. The experiments described here generally adhered to this expectation, as sensitivity and RT measures tended to be better in LSF conditions across the experiments, though independently of memory effects. It is worth noting that in the experiments where Bar elaborates his model of contextual guidance of object processing, the context, and familiarity with it, are mainly assumed. Indeed, other than in one experiment [20], the contextual association of the objects is determined by a questionnaire on a different set of participants, who classify the objects into ‘weak-’ and ‘strong-context’ categories. In the current set of experiments, context familiarity was controlled, and arbitrary associations were established between a given background contextual stimulus and a target location. Nevertheless, the discrepancy may stem from the very different natures of the tasks used. We hypothesized, based on previous findings that if LSFs drive the rapid recognition of objects, especially those with strong contextual associations, that in our experiment the targets with contextual memories would be selectively facilitated by LSFs as well. The fact that we did not observe this effect may be simply due to the fact that the ‘context’ in the Bar studies and in ours was of a different nature. In our experiments, they are specific, spatial-contextual long-term memories, perhaps episodic in nature, as opposed to familiar objects, embedded in a ‘schema’ of semantic associations, which may be processed in their contexts by a different set of neural structures in the MTL [46].

### Conclusions

The results from our experiments are more in line with theories that suggest that the differential contribution of spatial frequencies may be task dependent [45]. In the tasks described in this paper, both types of spatial frequencies may have aided in making a visual discrimination. Future studies will be needed to differentiate the effects of specific spatial frequencies in driving and/or aiding memory-guided attention in complex context-based visual search.

## Supporting Information

Table S1 Detailed F and p-values, and effect sizes for all conditions and interactions, across experiments 2–4.
(DOCX)
