# Supplementary material for: Is Attention Based on Spatial Contextual Memory Preferentially Guided by Low Spatial Frequency Signals?
Source: PLoS One. 2013 Jun 11;8(6):e65601. doi: 10.1371/journal.pone.0065601 (PMC3679178; doi:10.1371/journal.pone.0065601)
Supplement: Table S1 — Detailed F and p-values, and effect sizes for all conditions and interactions, across experiments 2–4. (DOCX) [file pone.0065601.s001.docx]

| **E2** |  |  | **F** | **p** | **η_p_^2^** |
| --- | --- | --- | --- | --- | --- |
|  | **d'** | sf | 5.390 | 0.008 | 0.200 |
|  |  | mem | 12.890 | 0.002 | 0.380 |
|  |  | sf x mem | 0.614 | 0.550 | 0.030 |
|  |  |  | | | |
|  | **RT** all conditions | sf | 16.850 | 0.000 | 0.450 |
|  |  | mem | 0.150 | 0.700 | 0.007 |
|  |  | key | 28.750 | 0.000 | 0.580 |
|  |  | sf x mem | 0.250 | 0.780 | 0.012 |
|  |  | sf x key | 1.500 | 0.230 | 0.070 |
|  |  | mem x key | 42.100 | 0.000 | 0.670 |
|  |  | sf x mem x key | 0.500 | 0.610 | 0.020 |
|  | **RT** target present only |  | | | |
|  |  | sf | 7.492 | 0.002 | 0.260 |
|  |  | mem | 14.357 | 0.001 | 0.410 |
|  |  | sf x mem | 0.431 | 0.650 | 0.020 |
|  |  |  | | | |
| **E3** | **d'** | sf | 2.780 | 0.120 | 0.160 |
|  |  | mem | 20.380 | 0.000 | 0.580 |
|  |  | isi | 0.149 | 0.710 | 0.010 |
|  |  | sf x mem | 0.284 | 0.600 | 0.020 |
|  |  | sf x isi | 0.170 | 0.700 | 0.010 |
|  |  | mem x isi | 0.050 | 0.830 | 0.003 |
|  |  | sf x mem x isi | 0.182 | 0.700 | 0.010 |
|  |  |  | | | |
|  | **RT** all conditions | sf | 19.790 | 0.000 | 0.570 |
|  |  | mem | 0.730 | 0.410 | 0.050 |
|  |  | isi | 16.130 | 0.001 | 0.520 |
|  |  | key | 35.260 | 0.000 | 0.700 |
|  |  | sf x mem | 0.008 | 0.930 | 0.001 |
|  |  | sf x isi | 1.200 | 0.290 | 0.075 |
|  |  | mem x isi | 0.080 | 0.780 | 0.006 |
|  |  | sf x mem x isi | 0.310 | 0.590 | 0.020 |
|  |  | sf x key | 0.190 | 0.670 | 0.013 |
|  |  | mem x key | 14.900 | 0.002 | 0.500 |
|  |  | sf x mem x key | 0.001 | 0.980 | 0.000 |
|  |  | isi x key | 0.150 | 0.700 | 0.010 |
|  |  | sf x isi x key | 0.060 | 0.800 | 0.004 |
|  |  | mem x isi x key | 0.030 | 0.860 | 0.002 |
|  |  | sf x mem x isi x key | 0.230 | 0.640 | 0.015 |
|  | **RT** target present only |  | | | |
|  |  | sf | 6.150 | 0.026 | 0.290 |
|  |  | mem | 4.263 | 0.057 | 0.220 |
|  |  | isi | 9.280 | 0.008 | 0.380 |
|  |  | sf x mem | 0.007 | 0.100 | 0.000 |
|  |  | sf x isi | 0.720 | 0.410 | 0.046 |
|  |  | mem x isi | 0.007 | 0.940 | 0.000 |
|  |  | sf x mem x isi | 0.630 | 0.440 | 0.040 |
|  |  |  | | | |
|  | | | **F** | **p** | **η_p_^2^** |
| **E4** | **d'** | sf | 3.028 | 0.097 | 0.130 |
|  |  | mem | 0.373 | 0.548 | 0.018 |
|  |  | sf x mem | 0.024 | 0.879 | 0.001 |
|  |  |  | | | |
|  | **RT** all conditions | sf | 9.040 | 0.007 | 0.310 |
|  |  | mem | 5.508 | 0.029 | 0.220 |
|  |  | key | 48.700 | 0.000 | 0.710 |
|  |  | sf x mem | 0.007 | 0.932 | 0.000 |
|  |  | sf x key | 0.029 | 0.870 | 0.001 |
|  |  | mem x key | 14.290 | 0.001 | 0.420 |
|  |  | sf x mem x key | 2.080 | 0.170 | 0.090 |
